# Reply to Hårdemark, B. Misconceptions Yield Misleading Results. Comment on “Wong et al. Comparative Study of Eclipse and RayStation Multi-Criteria Optimization-Based Prostate Radiotherapy Treatment Planning Quality. *Diagnostics* 2024, *14*, 465”

**DOI:** 10.3390/diagnostics15121494

**Published:** 2025-06-12

**Authors:** John Y. K. Wong, Vincent W. S. Leung, Rico H. M. Hung, Curtise K. C. Ng

**Affiliations:** 1 Department of Health Technology and Informatics, Faculty of Health and Social Sciences, The Hong Kong Polytechnic University, Hong Kong SAR, China; wongyukkwong2013@gmail.com; 2 Department of Clinical Oncology, Pamela Youde Nethersole Eastern Hospital, Hong Kong SAR, China; ricohung@connect.hku.hk; 3 Curtin Medical School, Curtin University, GPO Box U1987, Perth, WA 6845, Australia; curtise.ng@curtin.edu.au; 4 Curtin Medical Research Institute (Curtin MRI), Faculty of Health Sciences, Curtin University, GPO Box U1987, Perth, WA 6845, Australia

## 1. Introduction

Thank you for the opportunity to reply to the comment raised by Hårdemark [1] on our previous comparative study of multi-criteria optimization (MCO) implementations in commercial treatment planning systems [2]. We thank Hårdemark for the detailed and thoughtful critique. The points raised help clarify an important distinction between benchmarking strategies that prioritize methodological consistency versus those that emphasize clinical realism. Our study was designed as a systematic dosimetric comparative analysis that prioritized reproducibility and fairness by applying equivalent optimization objectives across both RayStation version 12A (RaySearch Laboratories AB, Stockholm, Sweden) and Eclipse version 16.1 (Varian Medical Systems, Inc., Palo Alto, CA, USA). The intent was not to replicate clinical optimization workflows but to isolate system performance under controlled, reproducible setups.

Hårdemark [1] asserted that the RayStation configurations for our original study were “incorrect”, but his comment confirmed that we did follow the RayStation user’s manual to devise our study methodology. In our direct correspondence, he also confirmed that he was able to replicate our findings by following the methodology described in our original study. Our study methods were devised based on established MCO research methodology [3] and the RayStation user’s manual [4], and standardized parameters were used for isolating system performance from planner variability. Nonetheless, we acknowledge Hårdemark’s [1] approach, which reflects a clinically oriented workflow, although the specific configuration referenced is based on non-peer-reviewed literature [5]. We agree that manuals are helpful starting points but may not fully capture the behavior of system functionalities.

## 2. Standardization in MCO Research

The importance of standardization in MCO comparisons is well-established in the peer-reviewed literature. Craft et al.’s work on Pareto surface generation explicitly demonstrates this principle through their use of objectives and constraints in analyzing tradeoffs between target coverage and organ-at-risk sparing [6]. In their prostate and skull base tumor case study, they employed clearly defined metrics including rectum mean dose, brainstem maximum dose, pituitary maximum dose, etc., and planning target volume dose as tradeoff settings, recognizing that such standardization is necessary for reproducible analysis of system capabilities.

Our study adopted that same approach by implementing identical optimization endpoints in both Eclipse and RayStation systems. We selected the rectum D50 ≥ 50 Gy objective as a standardized benchmark that could be equally applied to both systems. This method allowed us to isolate and compare the inherent optimization performance between the systems, free from the confounding effects of planner-specific tuning. We acknowledge the concern that the selected objectives may fail to reflect realistic optimization behavior. The objective value was used as a trade-off anchor to ensure parity across systems, not to impose a clinical constraint. Nonetheless, we accept that this may have inadvertently restricted RayStation’s potential. We recognize that a more clinical trade-off function—such as a descending equivalent uniform dose (EUD) or an unconstrained minimum objective—might provide a better demonstration of RayStation’s capabilities.

## 3. The Problem with Clinical Optimization in Comparative Studies

Hårdemark’s comment [1] suggested that we should employ more aggressive optimization techniques, including unbounded minimization and EUD objectives. While these approaches may improve plan quality in clinical settings, we believed they would compromise the study’s reproducibility across platforms.

Craft et al.’s work on MCO clearly demonstrated the limitations of subjective optimization approaches and emphasized that “a good set of relative weighting factors... must be found by the treatment planner in a time-consuming iterative manner [6].” While this warning was originally framed in the context of advocating for MCO planning in clinical practice, this also indicated that clinical fine-tuning would introduce non-reproducible factors in our dosimetric comparison study. We adopted fixed parameters across all cases to minimize this variability and highlight intrinsic system differences. However, we appreciate that demonstrating system capabilities under typical usage is a more informative benchmark for real-world performance, and future work should consider hybrid approaches that combine standardization and clinically representative configurations.

## 4. Conclusions

We appreciate the constructive discussion initiated by Hårdemark [1], which highlights an important distinction between benchmarking strategies that emphasize methodological standardization and those that aim to reflect clinical realism. Our study followed the former approach to ensure reproducibility and control across the systems; however, we recognize that this comes with limitations. We advise readers to interpret the findings of our original paper with caution, particularly in regard to RayStation’s full optimization capabilities under routine clinical use.
